# Effect of the Sorption Layer on the Protection Time Provided by Anti-Smog Half-Masks

**DOI:** 10.3390/ma16031230

**Published:** 2023-01-31

**Authors:** Agnieszka Brochocka, Oliwia Owczarek, Mateusz Wojtkiewicz

**Affiliations:** Department of Personal Protective Equipment, Central Institute for Labour Protection—National Research Institute, ul. Czerniakowska 16, 00-701 Warszawa, Poland

**Keywords:** anti-smog half-mask, activated carbon, protection time, respiratory system protection

## Abstract

This paper presents the results of a study examining the protection time of half-masks containing different types and quantities of carbon sorbents. The protection time afforded by the half-masks was determined by analyzing the adsorption of three substances harmful to human health at their maximum allowable concentrations. Two of the tested half-masks showed comprehensive protection against pollutants contained in smog. Among the tested half-masks, the one with the best protective properties was identified. The longest protection time (over 8 h) was recorded for toluene, followed by over 7 h for sulfur dioxide, and only 2 h for cyclohexane. The morphological structure of nonwovens incorporating the carbon sorbents was examined under a microscope. The study showed that protection time depends on the size of activated carbon particles incorporated in the nonwoven structure as well as on their distribution. Based on these results, we identified the most effective morphological structure of the sorbent in the nonwovens.

## 1. Introduction

For many years now, scientists have been warning against the harmful effects of air pollution. Smog is a term referring to polluted fog containing particulate matter (PM10, PM2.5), harmful substances (nitrogen compounds, hydrocarbons, carbon oxides, chloride, sulfur compounds), as well as biological factors (odors resulting from waste decomposition) [1,2]. According to the World Health Organization (WHO), in 2019 the worst air quality was found in Central Africa, the Middle East, India, and China [3]. In the same year the European Environment Agency reported 307,000 premature deaths attributable to the inhalation of fine dust from the air, 40,400 deaths due to nitrogen dioxide overexposure, and another 16,800 deaths due to excessive exposure to ozone in the European Union member states. Mortality statistics indicate that particulate matter (PM) suspended in the air constitutes the gravest danger to human health. The maximum allowable concentrations (MAC) of particulate matter in atmospheric air are expressed in µg/m^3^ and are much lower than the corresponding MAC values for indoor workplaces. The greatest damage to human health is done by particles below 2.5 μm in diameter (PM2.5), as due to their small size they can penetrate the lungs and get into the bloodstream, increasing the risk of lung cancer and circulatory diseases [4]. In addition, the latest research has shown that viruses and microorganisms may adsorb to particles with a high area-to-volume ratio [5,6], which implies that the risk of infection by those microbes may increase with increased PM concentration in the air. Despite the fact that the annual MAC for PM2.5, set by the WHO at 25 µg/m^3^, was met in 2021, mortality statistics did not change much relative to 2019. Indeed, to halve the mortality rate attributable to the presence of particulate matter in the air, PM2.5 would have to be brought down to as little as 5 µg/m^3^ [7]. Currently, air is considered to be very high quality for PM2.5 ranging from 0 to 13 µg/m^3^ [8]. It should be noted that the MAC for PM2.5 is more than twice the level that would tangibly decrease mortality. Generally speaking, the concentrations of particulate matter and chemical compounds measured in atmospheric air do not exceed the standards adopted for workplace environments. Thus, employers are not obliged to provide any additional respiratory protection to workers. The above notwithstanding, humanity should strive to ensure optimum air quality. However, whenever air pollution levels exceed the relevant standards, respiratory protection devices should be used. The simplest means of protection against smog are filtering half-masks conforming to the health requirements stipulated in Regulation (EU) 2016/425 of the European Parliament and of the Council of 9 March 2016 [9]. Air pollution also necessitates measures aimed at protecting the entire population. Individuals exposed to smog for a prolonged time, whose health is at a particular risk due to respiratory or circulatory conditions or allergies, should use filtering half-masks (with an exhalation valve) bearing the CE mark to confirm that they comply with the binding requirements in terms of protection against aerosols (particulate matter, smoke, and mist) as well as against the vapors and gases that are found in smog below their MAC levels.

Anti-smog half-masks consist of an assembly of materials providing protection against smog pollutants. They are usually made of a structural nonwoven, an air-purifying nonwoven (filtering and absorbing or only absorbing pollutants), as well as a filtering melt-blown nonwoven. Pollutants are retained on the nonwoven surface by four mechanisms: inertial impaction, interception, Brownian diffusion, and electrostatic attraction [6]. This last mechanism is found in nonwovens subjected to electrostatic activation, which is done on an industrial scale by corona discharges and triboelectric charging with water (which has been gaining in popularity since 2020) [10,11]. Filtration efficiency can also be improved by the incorporation of different modifiers in the nonwoven structure, e.g., to improve rheological properties, enhance the electric charge, or impart bactericidal properties. Finally, activated carbon can be used to increase the sorption capacity of the nonwoven. The absorbing properties depend on the textural parameters of the activated carbons, i.e., volume, surface area, the distribution of micro- and macropores, average pore width, and BET surface area. Carbon sorbents are commonly used materials in absorbent respiratory protective equipment for protection against vapors and gases [12]. They are modified with activating agents, which can be physical or chemical, which affects the textural, structural and surface chemical properties of carbon sorbents and directs the adsorption processes towards specific chemicals [13]. W. Li et al. produced high-surface-area activated carbons from coconut shells using physical (vapor) activation. Carbonization temperature had a large impact on the efficiency and development of pores in the obtained biochar [14]. An activated coconut shell carbon was modified with a calcium solution and given a highly porous structure. This material is used in filtering half-masks to reduce carbon monoxide and hydrocarbon emissions [1]. The influence of a high temperature during the production of biochar was also shown by the authors of other works [15,16], who obtained biochar with a large surface area and numerous functional groups, which can be used to reduce the emission of gases and odor compounds. Based on the literature data, it can be seen that various types of carbon materials can be used for the adsorption of vapors and gases from the air.

The sorption capacity of activated carbon primarily depends on its porosity. Chemical compounds are mostly adsorbed inside micro- and mesopores, while macropores serve only as ducts transporting them to the adsorbing pores [1]. Adsorption on the surface of a sorbent may occur via Van der Waals forces (physical adsorption) or via interactions between molecules and chemical reactions (chemical adsorption). Activated carbon can be further impregnated by coating with certain chemical compounds (inorganic salts) to increase their chemisorption efficiency. A good case in point is activated carbon impregnated with zinc sulfate, which has high affinity for ammonia and its derivatives [12]. The properties of activated carbon vary depending on the raw material and production method. Different types of activated carbon differ in terms of micropore structure and active surface area, which affect their adsorption–desorption properties. Thus, it is necessary to select the right sorbent in terms of particle size distribution to be added to melt-blown nonwoven structures. The sorbent modifier is comminuted prior to addition. The properties of the resulting nonwoven will be affected by the type of activated carbon used and its treatment prior to incorporation in the nonwoven structure.

**Figure 1:**
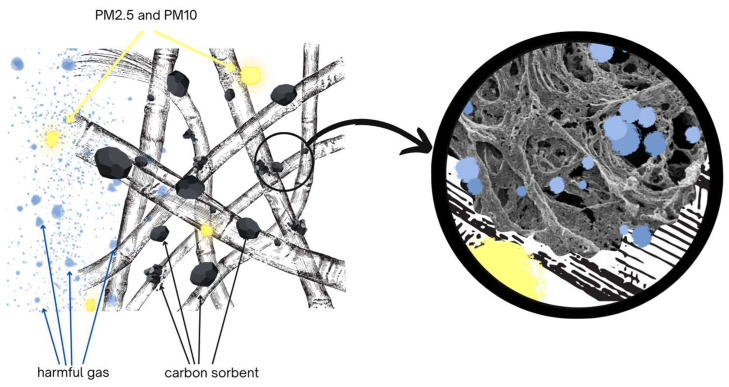
Mechanism of capturing pollutants by an anti-smog mask.

The paper compares the protective properties of different anti-smog filtering half-masks containing a sorption layer consisting of a carbon sorbent incorporated in a nonwoven. Half-masks maintain their protective properties against chemical vapors until the vapors penetrate the sorption bed. To evaluate the protection time provided by half-masks against harmful substances, the studied specimens were challenged with three test substances: cyclohexane, toluene, and sulfur dioxide. These substances and their derivatives typically occur in smog and constitute an increasing hazard to human health as air quality deteriorates. Cyclohexane itself is applied as a test substance in determining the minimum breakthrough time of sorbents challenged with organic vapors designated with the letter A pursuant to the standard EN 14387:2021. It is a nonpolar and nonreactive substance, due to which it primarily needs to be removed by physical adsorption [17]. According to literature data, the breakthrough time for cyclohexane vapors increases in proportion with the quantity of carbon sorbent in the nonwoven [9]. Toluene irritates the eyes of exposed individuals, is oto- and neurotoxic, and adversely affects reproduction. Sulfur dioxide belongs to harmful inorganic compounds. At small concentrations, it is not hazardous, but when released into the air by industrial processes and by coal combustion for energy purposes, it becomes one of the main compounds responsible for acid rains. Long-term exposure to sulfur dioxide damages the respiratory system and adversely affects the spleen, liver, lymph nodes, and brain [2]. Commercial filtering half-masks provide protection only against solid and liquid aerosols. Half-masks should have an absorbent layer with activated carbon in their structure to offer protection against the gases and vapors of chemical substances present in smog. The mechanism of operation of anti-smog half-masks is shown in Figure 1. Particulate matter ranging from PM2.5 to PM10 is retained on the elementary fibers of the filter fabric. On the other hand, gas and vapor molecules are captured by activated carbon particles due to their developed surface and high porosity. Harmful molecules are retained in the micro- and macropores of the sorbent.

Finished products with carbon sorbents were tested in this work, and therefore the authors did not have the material required for the determination of the chemical composition and reactions taking place on the surface of the sorbent. In order to compare the morphological structure of the activated carbons used in different half-masks, the adsorbing nonwovens were observed under the microscope to evaluate the size and distribution of carbon particles as well as to check for the presence of particle agglomerations.

The objective of the study was to determine the protection time offered by anti-smog half-masks incorporating a nonwoven layer with activated carbon. For a half-mask to afford appropriate protection against aerosols and harmful gases present in smog, it should contain some filtering-adsorbing material characterized by appropriate protective and functional properties. Unfortunately, in reality the layers with activated carbon used by manufacturers of half-masks designed to protect against chemical substances present in smog very often do not exhibit protective properties. Effective protection against pollutants depends on selecting the appropriate type and quantity of carbon sorbent, on the method of sorbent incorporation in the nonwoven structure, and on sorbent treatment prior to its addition. Not every nonwoven with activated carbon will exhibit sufficient sorption properties, and it therefore may not effectively protect users against the harmful substances contained in smog.

## 2. Materials

The study involved anti-smog half-masks made of an outer structural spun-bond nonwoven with a fluorescent print, a filtering-adsorbing nonwoven with a surface density of 150 g/m^2^ containing 90 g of activated carbon, a filtering nonwoven, and an inner structural spun-bond nonwoven (50 g/m^2^). The filtering-adsorbing nonwoven was made using a melt-blown process from Borealis HL 508J granulated isotactic polypropylene (PP) (NEXEO Solutions Poland Sp. z o.o., Warsaw, Poland) to which AG Pleisch 30180 PL MC 12 × 20 CBRN carbon sorbent (PLEISCH, Bäretswil, Switzerland) was added. It has been shown that the comminution of carbon sorbent in a planetary mill does not adversely affect its textural properties [9]. In addition, three commercially available FFP2 filtering half-masks with a carbon sorbent were included in the study for the purpose of comparing protection times and morphological structures.

The study encompassed a total of four different models of half-masks incorporating activated carbon in the structure of nonwoven material, characterized in Table 1. The structure of the half-masks is described from the outer layer exposed to the inflow of polluted air to the inner layer on the user’s side. In the remaining part of the text, the half-mask models are designated with numbers as in Table 1.

Table 2 describes the protective and functional parameters of the studied half-masks. The tested specimens comply with the FFP2 requirements of the standard EN 149:2001 + A1:2009, as claimed by the manufacturer. Their filtering performance against sodium chloride aerosol and paraffin oil mist is not less than 94%. At the same time, the inhalation resistance of the half-masks is consistent with the first protection class (up to 210 Pa). Half-mask 3 was found to have superior protective and functional parameters as compared to the other specimens. This half-mask was the only one with the third protection class against paraffin oil mist (up to 1%) and with a mean total inward leakage of less than 1%.

## 3. Methods

### 3.1. Determination of Protection Time

Protection time is defined as the minimum time during which half-masks exhibit protective properties. The half-masks were challenged with cyclohexane (MAC = 81 ppm), toluene (MAC = 26 ppm), and sulfur dioxide (MAC = 0.5). Each half-mask was challenged with each test substance using the same test parameters and laboratory conditions. The sorbent is considered to provide protection until the sorption bed is penetrated by the test substance, which is the moment when the concentration of the substance exceeds its MAC value downstream of the specimen (implying loss of protection and exposure of the user to the harmful substance). Protection time depends on the capacity of the sorption bed, the concentration and flow rate of the harmful substance, the presence of other harmful substances, and the external conditions, such as temperature and humidity.

**Figure 2:**
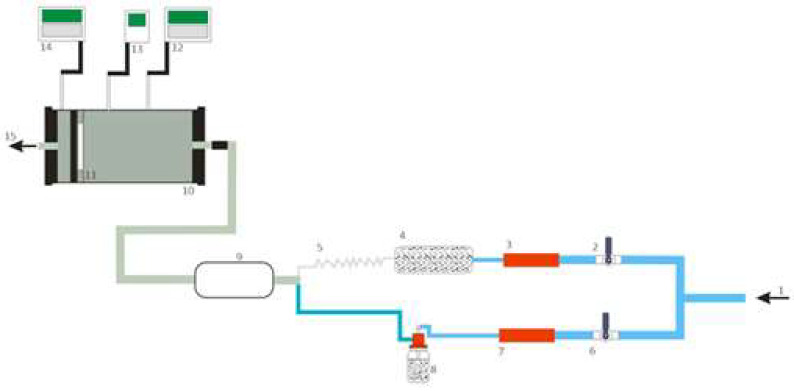
Experimental station for testing the protection time of half-masks against chemical substances: 1*—*inlet of dry compressed air; 2, 6*—*air valves; 3, 7*—*mass flow meters; 4*—*humidifier, 5*—*cooler; 8*—*evaporator; 9*—*test substance and air mixer; 10*—*pneumatic mount; 11*—*sample; 12, 14*—*gas detectors; 13*—*thermohygrometer; 15*—*test substance outlet to the fume hood. Source: [9].

The experimental station shown in Figure 2 has an air control system fitted with two Red-Y for Gasflow controllers. The system consists of two subsystems. Subsystem A is responsible for adjusting humidity and temperature, while subsystem B—for controlling substance concentration in the test chamber. Subsystem A supplies air to the humidifier, and subsequently to the cooler. In turn, subsystem B supplies compressed air to the evaporator with the test substance, which generates aerosol. The harmful test substance and pure air supplied by subsystems B and A are mixed in the mixing tank. The sample placed in the pneumatic mount is challenged with the resulting mixture. Two Dräger X-am 7000 gas detectors and analyzers record the concentration of the test substance upstream and downstream of the mounted sample. Throughout the test, the concentration of the test substance should not deviate by more than ±5 ppm. Tests were conducted at a volumetric flow rate of 30 L/min, relative humidity of (70 ± 5)% and a temperature of (21 ± 1) °C.

### 3.2. Microscopic Examination of Structural Properties

Samples taken from the adsorbing nonwoven layers of the studied half-masks were examined microscopically in terms of their morphological properties. Examinations were conducted using an Axiotech reflected light microscope (ZEISS, Jena, Germany). The morphological structure of activated carbon was observed at ×50 and ×100 magnifications. Special attention was paid to the quantity and distribution of carbon particles in the polymeric nonwoven structure.

## 4. Results and Discussion

### 4.1. Protection Time of Half-Mask

**Figure 3:**
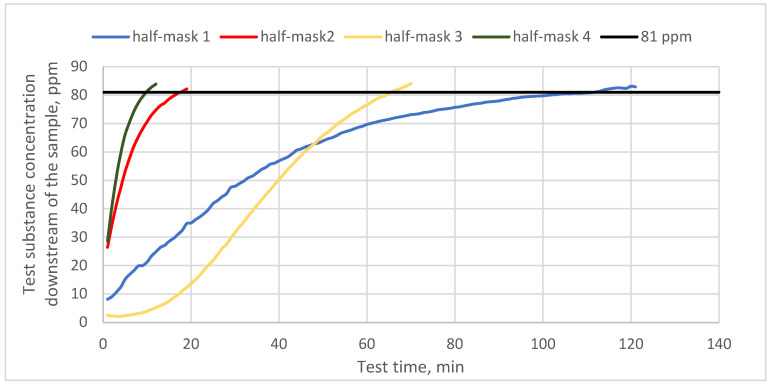
Protection time against cyclohexane for half-masks incorporating different types and quantities of carbon sorbents.

**Figure 4:**
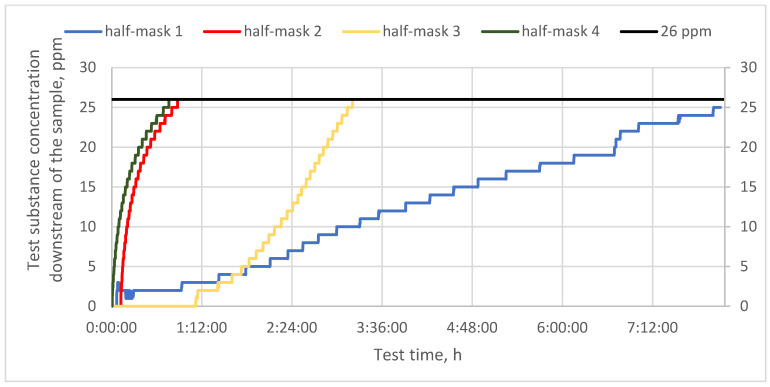
Protection time against toluene for half-masks incorporating different types and quantities of carbon sorbents.

**Figure 5:**
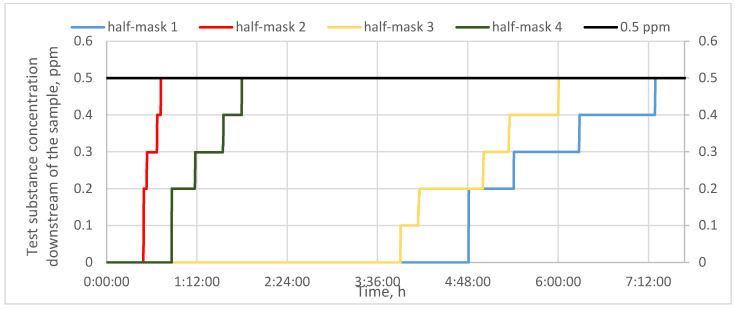
Protection time against sulfur dioxide for half-masks incorporating different types and quantities of carbon sorbents.

Figure 3 shows protection time results for half-masks containing different types and quantities of carbon sorbents challenged with cyclohexane vapors. In turn, Figure 4 and Figure 5 present protection time results for those half-masks challenged with toluene and sulfur dioxide vapors, respectively.

The obtained results show that protection time against cyclohexane vapors depended on the type and quantity of the sorbent. Each of the studied half-masks exhibited a different protection time against the test substance. The sorption properties of half-masks were tested at the MAC for cyclohexane (81 ppm). The shortest protection times were found for half-masks 2 and 4, with the sorption bed being penetrated by cyclohexane vapors after 19 and 10 min, respectively. Filtering half-masks 1 and 3 exhibited much longer protection times. Furthermore, the studied half-masks differed in terms of their initial response to cyclohexane. Half-mask 3 was characterized by the longest initial response and offered protection over nearly 70 min when challenged with cyclohexane. The longest protection time against the vapors of this test substance was found for half-mask 1 (100 min), despite a faster initial increase in the substance concentration downstream of the sample as compared to half-mask 3. The nonwoven layer with carbon sorbent from half-mask 1 was characterized by the highest surface density (360 g/m^2^). Similar results were obtained in the study by Franus et al. [18], who demonstrated that materials with activated carbon offer the best protection against organic substances. In their study, polymeric structures containing a carbon sorbent exhibited 53 min protection time against cyclohexane vapors. This is also consistent with the work of Das et al., who reported a satisfactory protection time against cyclohexane vapors for a carbon sorbent (50 min) [19].

In the next step, the studied filtering half-masks were challenged with toluene vapors. Measurements were conducted until the penetration of the sorption bed, that is, until a toluene concentration exceeding the MAC (26 ppm) was recorded downstream of the sample [20]. Protection time results for toluene vapors are given in Figure 4. In the case of half-masks 2 and 4, the sorption bed was penetrated after approx. 50 min. Half-mask 3 exhibited an intermediate protection time of about 2 h. Finally, a harmful concentration of toluene downstream of half-mask 3 was observed after 3 h from the beginning of the test. This half-mask also exhibited the slowest initial response among all the studied half-masks, as the concentration of toluene vapors downstream of the sample started to increase after more than 1 h of exposure. The sorption bed of half-mask 1 was not penetrated during the time of the experiment as after 8 h the downstream concentration of toluene vapors was 25 ppm. In this case, during the first hour, the concentration remained at 3 ppm, and subsequently increased by 1 ppm every 10–30 min. According to Ki-Hyun Kima et al. [21], activated carbon is the best material for the removal of airborne organic compounds such as benzene, toluene, and formaldehyde. In addition, given its regeneration and reuse properties, it is superior to other sorbents.

Finally, half-mask samples with carbon sorbents were challenged with sulfur dioxide. As can be seen from the results presented in Figure 5, the shortest protection time against this substance was found for half-mask 2 (40 min), while half-mask 4 offered a protection time of nearly 2 h. The protection times afforded by half-masks 1 and 3 were much longer: 6 and 7 h, respectively. In the case of half-mask 1, the first increment in the downstream concentration of sulfur dioxide vapors did not occur until 5 h of the experiment. These results are consistent with the study by Brochocka et al. [9], who reported that a filtering material incorporating activated carbon particles offered protection against sulfur dioxide for 6 h.

The measurements described above show that anti-smog filtering half-masks have different protection times against the studied test substances depending on the nonwoven containing a carbon sorbent. Each of the studied half-masks exhibited a different protection time for any given test substance. The shortest protection times against all three test substances were found for half-masks 2 and 4, in which the surface density of the adsorbing nonwoven was 350 g/m^2^ and 200 g/m^2^, respectively. In turn, the longest protection times against the three test substances were recorded for half-masks 3 and 1, with a surface density of the adsorbing nonwoven of 260 g/m^2^ and 360 g/m^2^, respectively. This shows that protection time is affected not only by the quantity of the carbon sorbent, but primarily by its quality, that is, its textural parameters. Indeed, according to the literature data, textural parameters including specific (BET) surface area are the basic measure of adsorption properties with reference to the sorption capacity of porous sorbents. The larger this capacity is, the longer the protection time it affords. An increase in the sorption properties of activated carbon has been reported by many authors [13,18,22,23].

### 4.2. Microscopic Examination of Structural Properties

**Figure 6:**
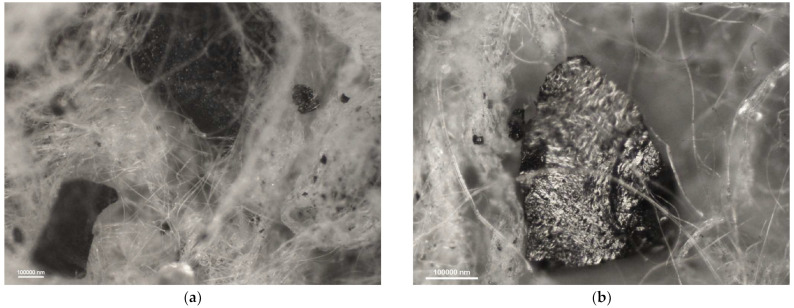
Photomicrographs of adsorbing nonwoven from half-mask 1 at a magnification of (**a**) ×50 and (**b**) ×100.

**Figure 7:**
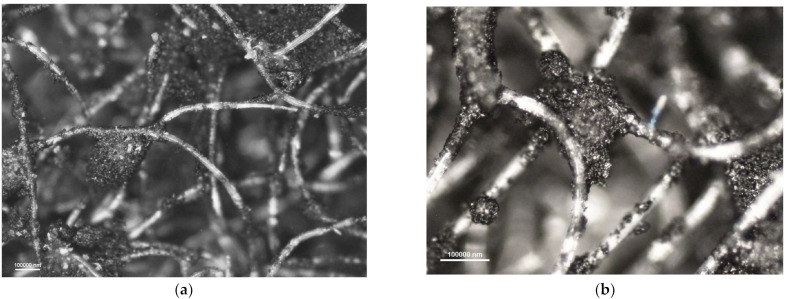
Photomicrographs of adsorbing nonwoven from half-mask 2 at a magnification of (**a**) ×50 and (**b**) ×100.

**Figure 8:**
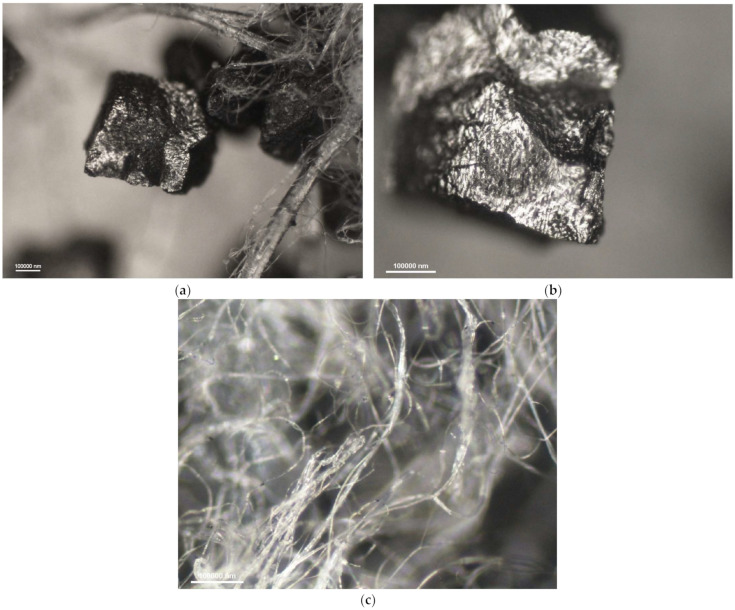
Photomicrographs of adsorbing nonwoven from half-mask 3 at a magnification of (**a**) ×50 and (**b**) ×100; (**c**) outer-layer nonwoven at a magnification of ×50.

**Figure 9:**
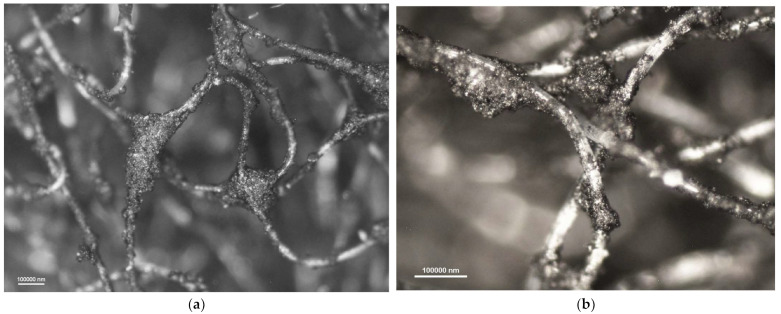
Photomicrographs of adsorbing nonwoven from half-mask 4 at a magnification of (**a**) ×50 and (**b**) ×100.

Figure 6, Figure 7, Figure 8 and Figure 9 show photomicrographs of nonwoven samples containing different types and quantities of carbon sorbents taken from half-masks 1–4.

The carbon sorbents in the studied nonwovens are characterized by irregular distribution. Activated carbon particles incorporated in the structure of the adsorbing nonwoven from half-mask 1 (Figure 6) are inhomogeneous in terms of size and are randomly distributed on the surface of fibers throughout the volume of the nonwoven. Particles smaller than 500 nm adhere to fiber surfaces, while larger particles either adhere to elementary fibers or occupy spaces between tangled fibers. The nonwovens from half-masks 1 and 3 are characterized by much thinner elementary fibers and larger carbon particles as compared to half-masks 2 and 4. The photomicrographs of adsorbing nonwoven samples with carbon sorbents from half-masks 2 and 4 (Figure 7 and Figure 9) showed them to be morphologically similar. In these two nonwovens, the carbon sorbent has the form of very fine particles adhering to elementary fibers or agglomerates completely filling some inter-filament spaces. It was difficult to examine these fibers under the microscope due to their silver color and light-reflecting properties. The nonwovens from half-masks 2 and 4 differ in terms of the density of elementary fibers in their structure. This property affects the size and quantity of carbon sorbent particles found between the tangled polymeric fibers (fibers in the nonwoven from half-mask 2 are more tangled). The nonwoven sample taken from half-mask 3, shown in Figure 8, exhibits the largest activated carbon particles, most of which have a diameter larger than that of a single PP fiber. In this half-mask, the sorbent layer is sandwiched between two filtering nonwoven layers forming a three-ply assembly (filtering nonwoven—carbon sorbent—filtering nonwoven). Here, activated carbon particles are found between elementary fibers. Figure 8 presents photomicrographs of activated carbon particles as well as an image of a nonwoven without a carbon sorbent modifier.

A comparison of adsorbing nonwovens indicates that those in half-masks 1 and 3 have much thinner fibers than those in half-masks 2 and 4. Analysis of photomicrographs revealed morphological similarity between the nonwovens of half-mask 2 and 4. Both exhibit very fine carbon sorbent particles of a similar size and with a similar pattern of distribution in the nonwoven structure. Each fiber in half-masks 2 and 4 seems to be covered with very small modifier particles. In addition, particle aggregates fill spaces between individual fibers.

When purchasing nonwovens incorporating carbon sorbents, the manufacturers of filtering half-masks should pay special attention to their sorption parameters, such as protection time against harmful substances. Filtering half-masks with sorption layers should not merely create an appearance of protection, but should rather protect their users according to their purpose, especially that such half-masks are also dedicated for urban residents (e.g., cyclists, joggers, the elderly and people with allergies) who wish to protect their respiratory system from smog pollutants at a time when the use of half-masks has become commonplace as a result of the COVID-19 pandemic.

## 5. Conclusions

The present analysis shows that not all commercially available filtering half-masks offer protection against all the air pollutants present in smog. Filtering half-masks with an EU certificate protect the respiratory system against aerosols, including particulate matter, smoke, and mist, but often not against gases and vapors [24]. The current study demonstrated that not every half-mask containing a layer with activated carbon can protect its users against the vapors of the harmful substances found in smog. The best protective properties against cyclohexane, toluene, and sulfur dioxide were recorded for a half-mask with an adsorbing nonwoven incorporating carbon sorbent particles of varied diameters, irregularly distributed throughout the volume of the nonwoven. As the aforementioned harmful substances are adsorbed only by activated carbon particles, the arrangement of nonwovens in the structure of a half-mask does not affect the protection time it provides. Sorption effectiveness mostly depends on the type and properties of the carbon sorbent used in the production of the adsorbing nonwoven. All the tested filtering half-masks meet the requirements of the standard EN 149:2001 + A1:2009 for the second protection class. However, only half-masks 1 and 3 provide comprehensive protection against smog components. These half-masks protect the respiratory tract against particulate matter, mists, smoke, as well as against harmful gas fumes.

## Figures and Tables

**Table 1:** Characteristics of the studied filtering half-masks.

Model no.	Photograph	Structural Elements of Half-Mask	Arrangement of Nonwoven Layers	Classification according to EN 149:2001 + A1:2009
1	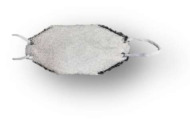	- flat foldable half-mask - no exhalation valve - no inner seal - external nose clip - ear loops - not adjustable	- structural spun-bond nonwoven with a surface density of 89 g/m^2^ - filtering-adsorbing nonwoven with a surface density of 360 g/m^2^ - filtering nonwoven with a surface density of 60 g/m^2^ - structural spun-bond nonwoven with a surface density of 80 g/m^2^	FFP2—second protection class (penetration up to 6%) NR—non-reusable
2	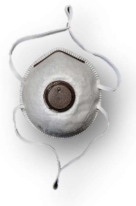	- cup-style half-mask - exhalation valve - inner nose seal - external nose clip - head straps - adjustable	- structural spun-bond nonwoven with a surface density of 40 g/m^2^ - filtering nonwoven with a surface density of 50 g/m^2^ - adsorbing nonwoven with a surface density of 350 g/m^2^ - nonwoven with a surface density of 70 g/m^2^ - needle-punched nonwoven with a surface density of 160 g/m^2^	FFP2—second protection class (penetration up to 6%) R—reusable D—clogging-resistant
3	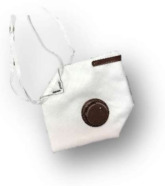	- flat foldable half-mask - exhalation valve - inner nose seal - external nose clip - head straps - adjustable	- two layers of structural spun-bond nonwoven with surface densities of 60 and 80 g/m^2^ - filtering-adsorbing nonwoven with a surface density of 260 g/m^2^ - filtering melt-blown nonwoven with a surface density of 20 g/m^2^ - structural spun-bond nonwoven with a surface density of 40 g/m^2^	FFP2—second protection class (penetration up to 6%) R—reusable D—clogging-resistant
4	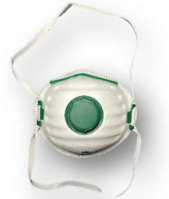	- cup-style half-mask - exhalation valve - inner nose seal - external nose clip - head straps - some limited adjustment	- needle-punched nonwoven with a surface density of 160 g/m^2^ - three layers of filtering melt-blown nonwoven with a combined surface density of 90 g/m^2^ - adsorbing nonwoven with a surface density of 200 g/m^2^ - needle-punched nonwoven with a surface density of 160 g/m^2^	FFP2—second protection class (penetration up to 6%) NR—non-reusableD—clogging-resistant

**Table 2:** Protective and functional parameters of half-masks with carbon sorbents.

Specimen	Paraffin Oil Mist Penetration, % (Flow Rate of 1.6 dm^3^s^−1^)	Sodium Chloride Penetration, % (Flow Rate of 1.6 dm^3^s^−1^)	Inhalation Resistance, Pa (Flow Rate of 1.6 dm^3^s^−1^)	Mean Total Inward Leakage, %
1	1.82	0.20	200.8	1.82
2	1.67	0.53	148.5	4.04
3	0.34	0.41	202.8	0.43
4	2.71	1.20	148.0	3.94

## Data Availability

The data presented in this study are available on request from the corresponding author.
